# Supplementary material for: Syndiotactic Polyolefins by Hydrogenation of Highly Stereoregular 1,2 Polydienes: Synthesis and Structural Characterization
Source: Polymers (Basel). 2024 Sep 25;16(19):2711. doi: 10.3390/polym16192711 (PMC11478897; doi:10.3390/polym16192711)
Supplement: Supplementary file 1 [file polymers-16-02711-s001.zip › polymers-3151579-supplementary.pdf]

Syndiotactic Polyolefins by Hydrogenation of Highly Stereoregular 1,2 Polydienes: Synthesis and Structural Characterization

Giovanni Ricci [1,*], Ivana Pierro [2] and Antonella Caterina Boccia [1]

[1] CNR-Istituto di Scienze e Tecnologie Chimiche "Giulio Natta" (SCITEC), Via A. Corti 12, I-20133 Milano, Italy; antonella.boccia@scitec.cnr.it
[2] Scientific Advisor, I-21052 Busto Arsizio (VA), Italy; ivanapierro@gmail.com
* Correspondence: giovanni.ricci@scitec.cnr.it

Supplementary materials

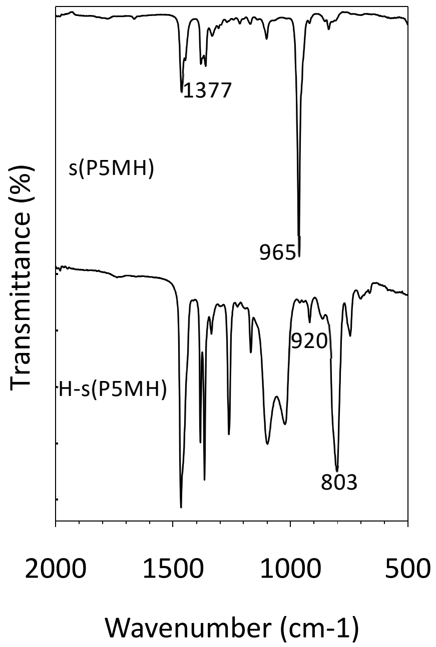

Figure S1. FTIR spectra of syndiotactc *trans*-1,2 poly(5-methy-1,3-hexadiene) (top, (**1,2^{sy}P5MHX**)) and its hydrogenated product syndiotactic poly(5-methyl-1-hexene) (bottom, [**H(1,2^{sy}P5MHX**)].

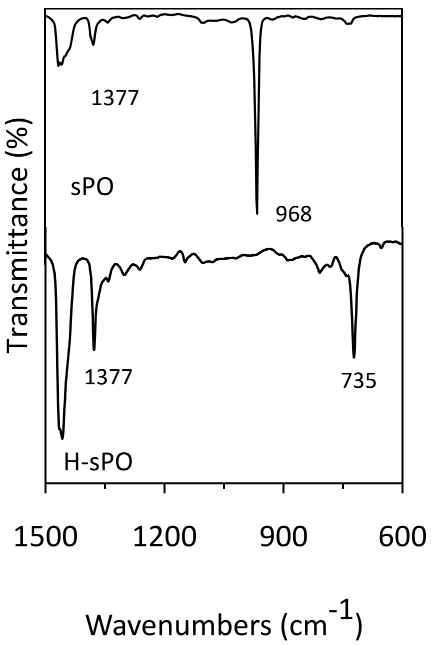

Figure S2. FTIR spectra of syndiotactic *trans*-1,2 poly(1,3-octadiene) **(1,2^{sy}PO)** (top) and its hydrogenated product syndiotactic poly(1-octene) (**[H(1,2syPO)]**, bottom).

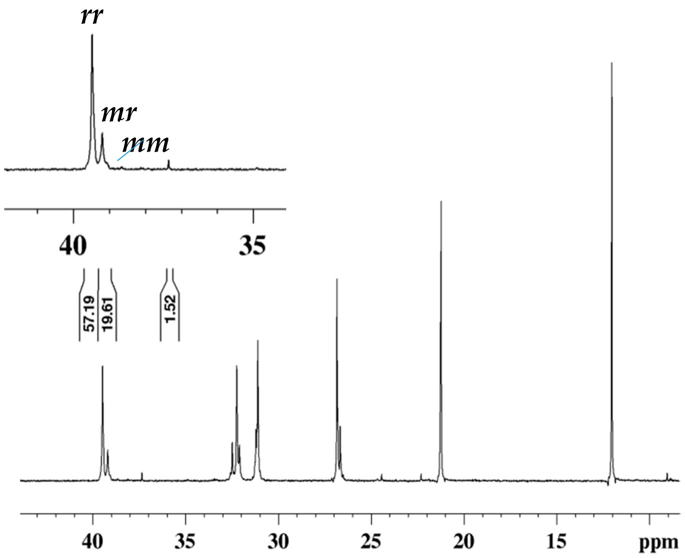

% tacticity = I [rr] / I [rr] + I [mr] + I [mm] x 100

% [rr] = 74,5%

Figure S3: ^{13}C NMR spectrum of **[H(1,2^{sy}PHX)]** sample @600 MHz in TCE, showing the assignment of tacticity at triad level.

Two-dimensional NMR parameter of acquisition

Two-dimensional heteronuclear ^{1}H-^{13}C experiments were acquired on a Bruker AVANCE DMX spectrometer operating at 600 MHz, (14.1 T), and 330 K. The g-HSQC experiment (gradient-heteronuclear single quantum correlation) was performed by applying a coupling constant $^{1}J_{CH}$ = 125 Hz; data matrix 2K × 256; number of scans:128; P1 as 90° pulse, was determined on each sample. The g-HMBC experiments, (gradient-heteronuclear multiple bond correlation), were performed by applying a delay of 100 ms for the evolution of long-range coupling; data matrix 2K × 512; number of scans 64; D1 2.00 s. Data were zero filled and weighted with a sine bell function before Fourier transformation. Differences of chemical shifts among mono- and two-dimensional experiments are due to the different temperature of data acquisition.

^{1}H-^{13}C HSQC (heteronuclear single quantum coherence) NMR experiment is used to determine proton-carbon single bond correlations.
^{1}H-^{13}C HMBC (heteronuclear multiple bond correlation) NMR experiment gives correlations between carbons and protons that are two and three bonds away

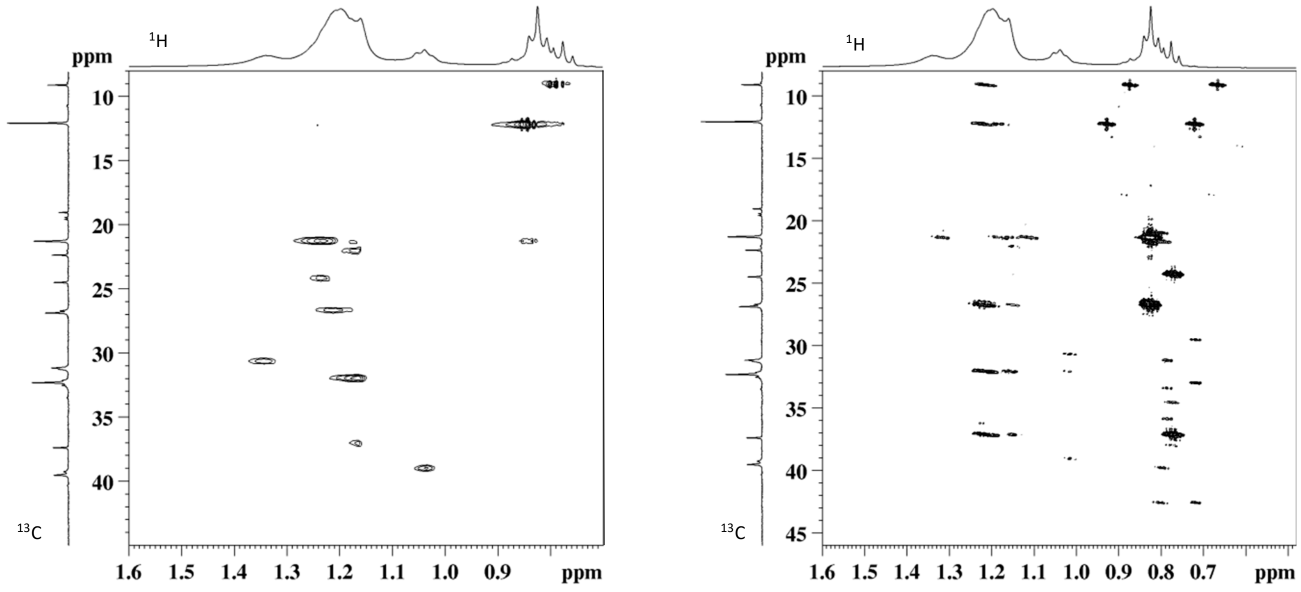

Figure S4: HSQC on the left) and HMBC (on the right) experiments of **[H(1,2^{sy}PHX)]** sample @600 MHz in TCE.

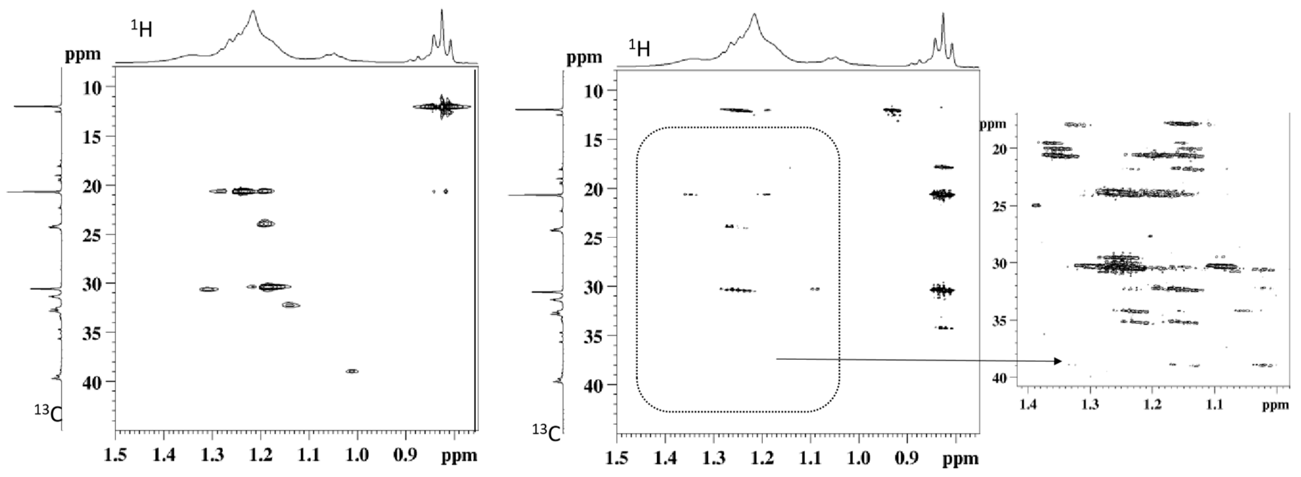

Figure S5: HSQC on the left) and HMBC (on the right) experiments of **[H(1,2^{sy}PHP)]** sample @600 MHz in TCE.
